# High Performance Poly(vinyl alcohol)-Based Li-Ion Conducting Gel Polymer Electrolyte Films for Electric Double-Layer Capacitors

**DOI:** 10.3390/polym10111179

**Published:** 2018-10-23

**Authors:** Jingwei Wang, Zejia Zhao, Shenhua Song, Qing Ma, Renchen Liu

**Affiliations:** 1 Shenzhen Key Laboratory of Advanced Materials, School of Materials Science and Engineering, Harbin Institute of Technology, Shenzhen 518055, China; hitjingweiwang@163.com (J.W.); wustmq@163.com (Q.M.); 2 State Key Laboratory in Ultra-precision Machining Technology, Department of Industrial and Systems Engineering, The Hong Kong Polytechnic University, Kowloon, Hong Kong, China; ze-jia.zhao@connect.polyu.hk; 3 Research Institute of Tsinghua University in Shenzhen, Shenzhen 518057, China; renchen.liu@gmail.com

**Keywords:** PVA, biodegradable gel polymer electrolyte, ionic conductivity, electric double layer capacitor, electrochemical properties

## Abstract

With 1-methyl-2-pyrrolidinone (NMP) as the solvent, the biodegradable gel polymer electrolyte films are prepared based on poly(vinyl alcohol) (PVA), lithium bis(trifluoromethane)sulfonimide (LiTFSI), and 1-ethyl-3 methylimidazoliumbis(trifluoromethylsulfonyl)imide (EMITFSI) by means of solution casting. The films are characterized to evaluate their structural and electrochemical performance. The 60PVA-40LiTFSI + 10 wt.% EMITFSI system exhibits excellent mechanical properties and a high ionic transference number (0.995), indicating primary ionic conduction in the film. In addition, because of the flexibility of polymer chain segments, its relaxation time is as low as 5.30 × 10^−7^ s. Accordingly, a high ionic conductivity (3.6 × 10^−3^ S cm^−1^) and a wide electrochemical stability window (~5 V) are obtained. The electric double-layer capacitor (EDLC) based on this electrolyte system shows a specific capacitance of 101 F g^−1^ and an energy density of 10.3 W h kg^−1^, even after 1000 charge-discharge cycles at a current density of 0.4 A g^−1^ under a charging voltage of 2 V. All these excellent properties imply that the NMP-soluble 60PVA-40LiTFSI + 10 wt.% EMITFSI gel polymer electrolyte could be a promising electrolyte candidate for electrochemical device applications.

## 1. Introduction

Nowadays, electric double layer capacitors (EDLCs) are one of the main types of energy storage device for sustainable development due to their advantages, such as shorter charge and discharge time, long cycle lifetime, and high power density [1,2,3,4]. Generally, liquid electrolytes are employed in the fabrication of these devices. Nevertheless, the fact remains that low electrolyte breakdown voltage (<1 V), solvent evaporation, or leakage of liquid electrolytes leads to some undesirable performance of supercapacitors [5]. By contrast, gel polymer electrolyte (GPE) films composed of a polymer matrix, salt, and plasticizer have received considerable attention owing to their fantastic performance, such as high safety, perfect flexibility, and high ionic conductivity [6]. Meanwhile, they are capable of replacing both the liquid electrolyte and separator in batteries or supercapacitors [7,8]. The reported polymer matrices of the GPE mainly include poly(vinylidene fluoride-co-hexafluoropropylene) (PVdF-HFP), poly(methyl methacrylate) (PMMA), poly(vinylpyrrolidone) (PVP), and poly(ethylene oxide) (PEO). The highest room-temperature ionic conductivity of the above GPEs is about 10^−5^–10^−3^ S cm^−1^ [9,10,11,12]. Apart from these one-component host polymers, some triblock copolymers and well-designed random copolymers have also been explored; for example, polystyrene-block-poly(methyl methacrylate)-block-polystyrene (PS-b-PMMA-b-PS) [13,14,15], and poly[styrene*-ran-*1-(4-vinylbenzyl)-3-methylimidazolium hexafluorophosphate] (P[S*-r-*VBMI][PF_6_]) [16]. These copolymers have high conductivity and perfect mechanical performance when they are used for electrochemical devices.

The aim of the present work is to use a biodegradable polymer to prepare gel polymer electrolyte films with high ionic conductivity and fantastic mechanical properties, which fulfill the requirements of global energy and the environment. Biodegradable poly(vinyl alcohol) (PVA) is mainly composed of vinyl alcohol groups, and their polar oxygen atoms could complex with the cations of dissolvable salts to form polymer electrolyte complexes. In addition, PVA has many outstanding advantages; for example, they are nontoxic and inexpensive, and have high charge storage capacity along with excellent mechanical properties [17]. However, PVA-based polymer electrolyte film for EDLC applications has rarely been reported. This could be owing to the fact that water is usually employed as a solvent of PVA, resulting in a high degree of crystallinity in the PVA electrolyte, and consequently, some negative impacts on ion transport in the polymer matrix. Hence, in the present study, 1-Methyl-2-pyrrolidinone (NMP) is employed as the solvent of PVA to prepare polymer electrolyte films, because NMP can act as a plasticizer for PVA, thereby increasing the amorphicity and flexibility of the electrolyte system [18]. Given the fact that Li-ion batteries and electric double layer capacitors can complement each other, and thus it is of importance to prepare Li-ion conducting electrolytes which can be applied both in batteries and supercapacitors, the biodegradable PVA-lithium bis(trifluoromethane)sulfonimide (LiTFSI) polymer electrolyte films were prepared and plasticized by 1-ethyl-3 methylimidazolium bis(trifluoromethylsulfonyl)imide (EMITFSI) to enable them to become gel polymer electrolyte films. In the PVA-LiTFSI-EMITFSI electrolyte system, TFSI^–^ and EMI^+^ are harmful to aquatic life, and thus this electrolyte is not completely environment-friendly, but it should be much better than non-biodegradable polymer host systems. Additionally, EMITFSI is safer than traditional plasticizers, such as ethylene carbonate (EC) and propylene carbonate (PC), and it can increase the number and mobility of charge carriers for EDLC applications. This study focused mainly on the effect of various amounts of LiTFSI or EMITFSI on the structural, electrical, and electrochemical performance of the NMP-soluble PVA-LiTFSI-EMITFSI polymer electrolyte films. The morphological and structural characteristics, mechanical properties, ionic conductivities, electrochemical stability windows, and ionic transference numbers of the films were investigated using scanning electron microscopy (SEM), X-ray diffraction (XRD), Fourier transform infrared spectroscopy (FTIR), mechanical testing, impedance spectroscopy, cyclic voltammetry, and DC (direct current) polarization.

Finally, the EDLCs were assembled with the optimized Li-ion conducting gel polymer electrolyte serving as both electrolyte and separator, and their performance was evaluated by cyclic voltammetry (CV) and galvanostatic charge-discharge (GCD) techniques, as well as powering a light-emitting diode (LED).

## 2. Materials and Methods

The original materials used mainly included PVA (Aldrich, *M*_W_ = 125,000 g mol^−1^, Shanghai, China), NMP (Aladdin, Shanghai, China), LiTFSI (Aladdin, Shanghai, China), EMITFSI (IoLiTec, Denzlingen, Germany), activated carbon (AC) (specific surface area: 1800–1900 m^2^ g^−1^, Kejingstar Technology, Shenzhen, China), poly(vinylidene fluoride) (PVdF, Kejingstar Technology, Shenzhen, China), and carbon black (Super P, Kejingstar Technology, Shenzhen, China).

The preparation method of the polymer electrolyte films followed literature [19]. In this work, the dried PVA was first dissolved in NMP by magnetic stirring at 80 °C for 5 h. Then different amounts of LiTFSI (10, 20, 30, 40, and 50 wt.%, denoted as P-10, P-20, P-30, P-40, and P-50, respectively) were mixed with the PVA solution and stirred continuously for several hours at the same temperature. To prepare gel polymer electrolyte films, different amounts of EMITFSI, that is, 5, 10, 15, and 20 wt.% EMITFSI, were added to the optimal LiTFSI-complexed PVA solution. If the optimized polymer electrolyte is the P-40 system, the gel polymer electrolyte systems would be denoted as P-40-5, P-40-10, P-40-15, and P-40-20. Finally, the transparent films with a thickness of approximately 90 μm (measured by a micrometer) were formed after evaporating the solvent NMP in a vacuum oven at 70 °C, and then stored in a glove box under an argon atmosphere.

The AC electrode was composed of 80 wt.% AC, 10 wt.% PVdF, and 10 wt.% super P, and the obtained viscous paste was doctor bladed on aluminum foil and dried at 110 °C for 12 h. The dried electrode was cut into a circular shape with a thickness of 30 μm and a mass loading of 0.0025 g.

The morphological characteristics of the electrolyte films were examined using scanning electron microscopy (Hitachi S-4700 SEM, Tokyo, Japan). The structural studies were carried out by a D/max Rigaku X-ray diffractometer (XRD, Tokyo, Japan) with Cu Kα radiation (λ = 1.5406 Å) in the 2*θ* range of 5° to 55°. The ion pairs in the electrolyte were analyzed using Fourier transform infrared spectroscopy (FTIR, Nicolet 380, Madison, WI, USA). The mechanical properties of the films with a size of 0.09 mm × 10 mm × 80 mm (thickness × width × gauge length) were measured using a universal testing machine (CMT7504, Ningbo, China) at room temperature under a crosshead speed of 10 mm/min. The electrochemical performance of the films, including both the electrochemical stability window and the total ionic transference number, were examined using cyclic voltammetry and DC polarization, respectively, by virtue of an electrochemical work station (Model: CHI760D). The cyclic voltammetry measurements were performed in the potential range of −3 to 3 V with a symmetrical two-electrode configuration (SS/electrolyte/SS). In the DC polarization study, a fixed 0.5 V DC voltage passed through the SS/electrolyte/SS cell and the obtained current was monitored as a function of time. The ionic conducting properties of the electrolyte films were evaluated via an AC (alternating current) impedance spectroscopy analyzer (PSM 1735, Newton, UK) in the frequency range of 10 MHz to 100 Hz with a signal level of 10 mV. The impedance measurements were conducted by sandwiching a film piece between two symmetrical stainless steel electrodes.

The AC/GPE/AC supercapacitor was charged and discharged at the operating potential ranges of 0–1.6 V, 0–2.0 V, 0–2.4 V, and 0–3.0 V. The galvanostatic charge-discharge (GCD) tests were conducted on a Neware testing system (Neware, Shenzhen, China) at room temperature. The cyclic voltammetry (CV) was performed with an electrochemical work station between 0 and 2.0 V at different scan rates.

## 3. Results and Discussion

**Figure 1:**
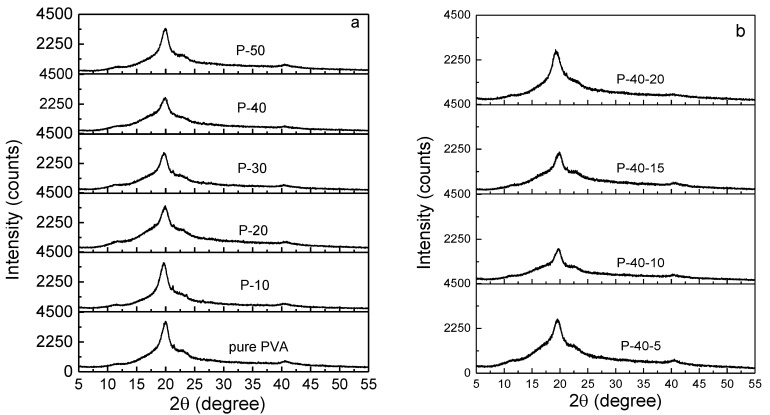
XRD patterns of (**a**) pure poly(vinyl alcohol) (PVA), P-10, P-20, P-30, P-40, and P-50; and (**b**) P-40-5, P-40-10, P-40-15, and P-40-20 films.

The polymer electrolyte film should exhibit more amorphous domains on account of the fact that the ion mobility increases as the amorphous domains rise. The XRD pattern of pure PVA film (see Figure 1a) indicates that it is a semicrystalline polymer. It shows four peaks at the 2*θ* angles of 11.2°, 19.9°, 21.3°, and 40.5°, and their crystal planes are identified as (100), (101), (1$\bar{0}$1), and (111), respectively [17]. There are no signals of the new peaks present after the addition of LiTFSI (see Figure 1a), implying that the salt could be complexed in PVA. Furthermore, with increasing LiTFSI content up to 40 wt.%, the widths of XRD peaks gradually increase, and the relative intensities decrease. In addition, the crystalline peak at 21.3° disappears, indicating the degree of crystallinity of the PVA polymer electrolyte film decreases; that is, the quantity of amorphous phase increases. This may be due to the complexation between LiTFSI and PVA, thereby preventing the interaction of the intermolecular and intramolecular force of PVA. As shown, the optimal addition of LiTFSI is 40 wt.%. Figure 1b represents the XRD patterns of 60PVA-40LiTFSI + *x* EMITFSI (*x* = 5, 10, 15, and 20 wt.%) films. Similarly, with increasing EMITFSI content up to 10 wt.%, the relative XRD intensities decrease and then progressively increase, and no new peak appears; the crystalline peak at the 2*θ* angle of 11.2° vanishes for the P-40-10 system. This implies that the ionic liquid could dissolve into the PVA + 40 wt.% LiTFSI system and disturb the intermolecular or intramolecular force of PVA (or both), resulting in an increase in the amorphous domains. However, as the amount of EMITFSI is increased to 15 wt.%, the relative peak intensity strengthens again; that is, the amorphous domains decrease again. This may be attributed to the ion aggregates between redundant anion TFSI^−^ and cation Li^+^ ions, resulting in a decrease in the amorphous domains of PVA.

**Figure 2:**
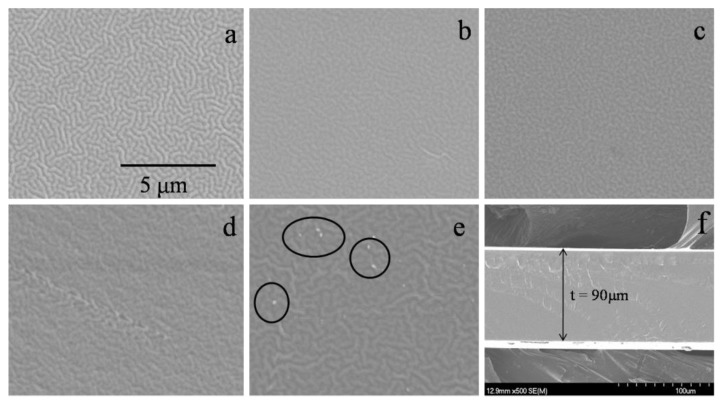
SEM images of different films: (**a**) pure PVA, (**b**) P-40, (**c**) P-40-5, (**d**) P-40-10, (**e**) P-40-15, and (**f**) cross-section of P-40-10.

The morphologies of pure PVA and P-40-*x* (*x* = 0, 5, 10, and 15 wt.%) polymer electrolyte films are represented in Figure 2. It is seen that all the electrolyte films exhibit a homogeneous and compact wrinkled texture due to the disorder structure of the polymer matrix. The wrinkled texture becomes apparent and soft fractal at the same magnification with increasing EMITFSI content up to 10 wt.% (see Figure 2d), but the microstructure of the films is still compact (see Figure 2f), indicating that the ionic liquid could disturb the ordered structure of PVA chains. However, when increasing EMITFSI content to 15 wt.%, some white granules appear as shown with circles in Figure 2e, which may be attributed to ion aggregates. These ion aggregates should be mainly the combination of Li^+^ and TFSI^−^ ions, but not EMI^+^ and TFSI^−^ ions. This is because the concentration of EMITFSI is low and the ionic force of EMITFSI is weak, and hence the surplus TFSI^−^ anions will combine with Li^+^ ions. Accordingly, the optimal amount of EMITFSI is around 10 wt.%. 

**Figure 3:**
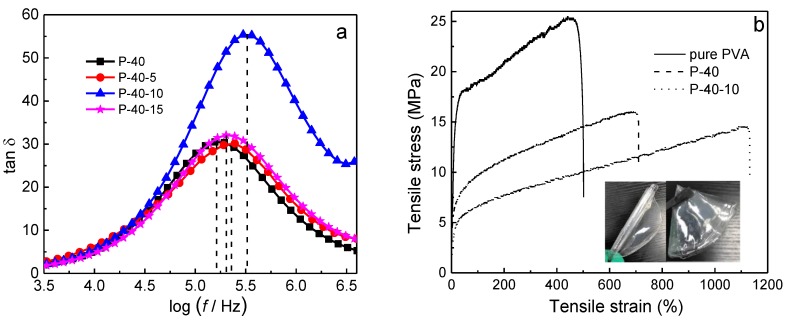
(**a**) Variation of loss tangent with frequency for P-40, P-40-5, P-40-10, and P-40-15. (**b**) Stress-strain curves of pure PVA, P-40, and P-40-10 films (insets show photographs of the films) recorded at room temperature.

Polymer chains may transit to one equilibrium from another under external force, accompanying elastic deformation due to polymer segmental movement. This segmental movement will need some time because of the internal friction among polymer segments. Relaxation time is a measure of how long it takes to achieve elastic deformation. Hence, the relaxation time is related to the flexibility and amorphous domains of polymer chain segments; that is, the lower the relaxation time, the higher the ionic conductivity is [20]. The variation of loss tangent with frequency for 60PVA-40LiTFSI + *y* EMITFSI (*y* = 0, 5, 10, and 15 wt.%) is shown in Figure 3a. As can be seen, the frequency corresponding to the peak shifts toward higher frequencies with increasing EMITFSI addition up to 10 wt.%. However, it shifts a little back to lower frequencies with further increasing EMITFSI addition. The relaxation time can be obtained by [21]

(1) ωτ=1 

where *ω* is the angular frequency at the peak, and τ is the relaxation time. The obtained relaxation times are listed in Table 1. Obviously, it is the shortest (5.30 × 10^−7^ s) for the P-40-10 system, implying that this system contains the most amorphous domains, being consistent with the XRD results. It could be anticipated that this system would have a high ionic conductivity.

Ideally, a polymer electrolyte film should also have outstanding mechanical properties in addition to excellent ionic conductivity for its practical applications. The typical stress-strain curves of pure PVA, PVA + 40 wt.% LiTFSI, and 60PVA-40LiTFSI + 10 wt.% EMITFSI gel polymer electrolyte films are shown in Figure 3b, with the photographs of the films as insets. Evidently, all the plots show similar characteristics, including the elastic behavior and plastic deformation. The obtained mechanical capacities of the films, such as Young’s modulus, breaking strain, and yield strength, are summarized in Table 2. With the addition of LiTFSI and EMITFSI, the Young’s modulus and yield strength progressively drop, but the breaking strain gradually increases, being 500% and 1130% for the pure PVA and P-40-10 systems, respectively. This implies that the flexibility and amorphicity of the films has considerably been enhanced, which is in favor of close electrolyte-electrode contact and ion migration. To illustrate once again, the photographs of the frizzled and folded P-40-10 films are shown in Figure 3 as insets. On the other hand, the values of Young’s modulus and yield strength are 26.6 and 6.2 MPa for the film, being enough for Li-ion device applications.

**Figure 4:**
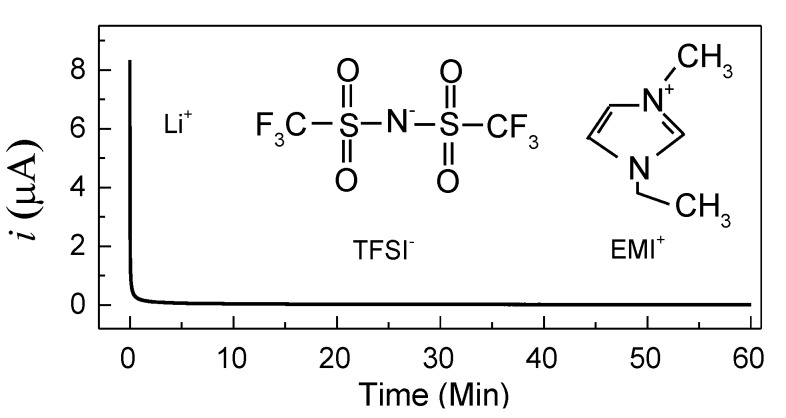
Direct current (DC) polarization plots of the SS/P-40-10/SS cell, recorded at room temperature under a voltage of 0.5 V (inset shows the chemical structures of TFSI^−^ and EMI^+^).

It is worth noting that a polymer electrolyte film should be an ionic conductor for Li-ion device applications. Hence, the ionic transference number (*t*_ion_) of cell-1 (SS/PVA + 40 wt.% LiTFSI/SS) has been evaluated by Wagner’s DC polarization at room temperature under a voltage of 0.5 V. The polarization plots are shown in Figure 4 with the chemical structures of TFSI^−^ and EMI^+^ as insets. The value of *t*_ion_ is given by [22]

(2) $$t_{\mathrm{ion}}=\frac{i_{I}-i_{F}}{i_{I}}\;$$

where *i*_I_ is the initial current including both ion and electron conductions, and *i*_F_ represents the final steady-state current including just electron conduction. According to Equation (2), the ionic transference number is 0.995, which is similar to previous reports. For example, Polu et al. [22,23] reported that the ionic transference numbers of PVA-Mg(CH_3_COO)_2_ and PVA-Mg(NO_3_)_2_ are 0.96 and 0.98, respectively. Apart from the high ionic transference number, the current falls rapidly with time, indicating this electrolyte is an ionic conductor [24]. Furthermore, it is worth noting that three types of ions (Li^+^, TFSI^−^, and EMI^+^) are available for conduction in this electrolyte system, but Li^+^ is far smaller than both TFSI^−^ and EMI^+^, which may be firmly trapped by PVA chains. Consequently, Li^+^ may migrate faster than other large-sized ions in the PVA matrix, making a greater contribution to the ionic conduction [25,26].

**Figure 5:**
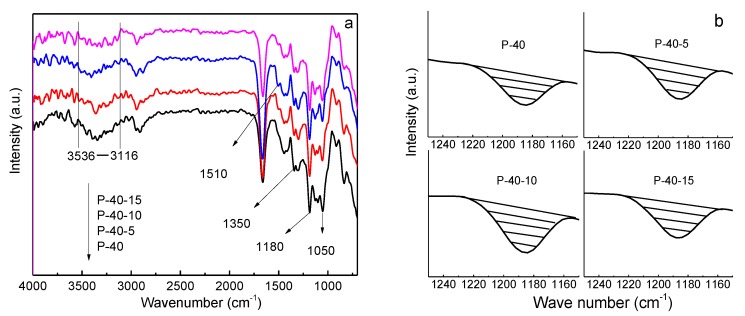
FTIR spectra of P-40, P-40-5, P-40-10, and P-40-15 films in the ranges of (**a**) 4000–700 cm^−1^ and (**b**) 1250–1150 cm^−1^.

The transport of Li-ions mainly occurs in the amorphous regions of PVA by coordinating with –OH and migrating with PVA segmental movements under an electric field, which is similar to the transport of Li-ions in PEO [27,28], and can be analyzed through FTIR. The FTIR spectra of P-40-*x* (*x* = 0, 5, 10, and 15) films are shown in Figure 5. As can be seen in Figure 5a, for the P-40 system, the absorption band of –OH is in the range of 3536–3120 cm^−1^, and it shifts to the left gradually for the P-40-5 and P-40-10 systems, but returns back to the right for the P-40-15 system, indicating that more Li-ions interact with –OH in the P-40-10 system as compared with the other systems. The number of transporting Li^+^ ions in the electrolyte film can also be evaluated through the amount of TFSI^−^, which is proportional to its characteristic peak area shown in the FTIR spectra (Figure 5b); that is, the bigger the area is, the more free TFSI^−^ ions exist, and thus the more dissociated Li^+^ ions there are interacting with PVA. The peaks at 1050, 1183, and 1350 cm^−1^ correspond to the characteristic peaks of TFSI^−^, and the new peak at 1510 cm^−1^ is probably assigned to EMI^+^, as shown in Figure 5a. Considering the fact that the ionic association between Li^+^ and TFSI^−^ ions could occur at the sulfamide functional group (R–SO_2_–N< at 1180 cm^−1^) end of TFSI^−^, the peak areas at about 1183 cm^−1^ of P-40-*x* (*x* = 0, 5, 10, and 15) films are depicted in Figure 5b. The peak area increases with rising EMITFSI concentration until 10 wt.%, and then falls with further addition of EMITFSI. This means that the 60PVA-40LiTFSI + 10EMITFSI system has more TFSI^−^ and dissociated Li^+^ ions; that is, this system contains a higher concentration of mobile ions, which is in agreement with the results shown in Figure 5a.

**Figure 6:**
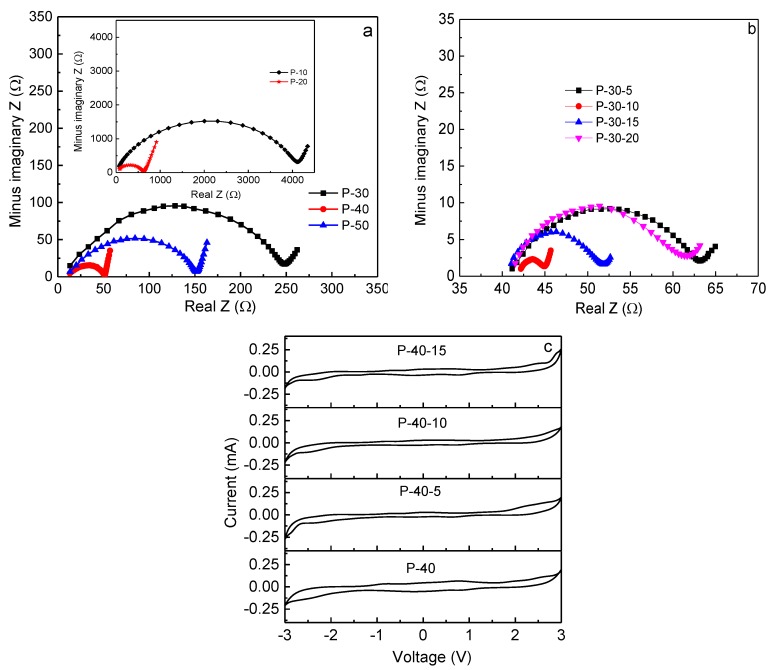
Nyquist impedance plots of (**a**) P-10, P-20, P-30, P-40, and P-50 films; and (**b**) P-40-5, P-40-10, P-40-15, and P-40-20 films. (**c**) Cyclic voltammograms for P-40, P-40-5, P-40-10, and P-40-15 films.

Generally, the ionic conductivity of an electrolyte membrane is described by [25]

(3) $$\sigma =\sum n_{i}q_{i}\mu_{i}\;$$

where *n*_i_ stands for the concentration of mobile ions, *q*_i_ represents the charge of mobile carriers, and *µ*_i_ represents the carrier mobility. In our study, *q*_i_ is the same for all samples. Additionally, the ionic conductivity (σ) of the electrolyte membrane can be calculated by

(4) $$\sigma =\frac{t}{R_{b}A}\;$$

where *t* stands for the film thickness, *R*_b_ denotes the bulk resistance, and *A* is the film-electrode contact area. *R*_b_ can be accurately measured using impedance spectroscopy. The Nyquist impedance plots of PVA + *x* LiTFSI (*x* = 10, 20, 30, 40, and 50 wt.%) and 60PVA-40LiTFSI + *y* EMITFSI (*y* = 5, 10, 15, and 20 wt.%) films are shown in Figure 6a,b, respectively. All the films show a typical gel polymer electrolyte impedance pattern, with a single semi-circular arc at high frequencies, and an inclined line at low frequencies [29]. Obviously, the circular arc progressively shrinks with increasing LiTFSI content up to 40 wt.% and then expands, implying that the bulk resistance of the film decreases and then increases, or the ionic conductivity increases and then decreases. This is attributed to the greater quantity of mobile Li^+^ ions that are available in the 40 wt.% LiTFSI film to coordinate with PVA, and then hop from one site to another. Upon the addition of EMITFSI, the circular arc continues to shrink due to the ionic liquid dissolution within the PVA matrix, and mainly acts as a plasticizer, which makes the polymer chains more flexible and the amorphous domains expand, resulting in an increase in ion mobility *µ*_i_. With the addition of EMITFSI to 10 wt.%, the room-temperature ionic conductivity of the film reaches a peak value, since the excess ionic liquid makes ions aggregate, leading to a decrease in the concentration of mobile ions *n*_i_. Furthermore, the intermolecular and intramolecular interactions in PVA could be enhanced, and then the amorphous domains would shrink, which is in accordance with the results of XRD and SEM. For the P-40-10 system, the room-temperature ionic conductivity is ~3.6 × 10^−3^ S cm^−1^, which is high enough for Li-ion device applications. So far, the reported room-temperature ionic conductivity of gel polymer electrolyte films is in the order of 10^−5^–10^−3^ S cm^−1^ [9,30,31,32,33,34].

The electrochemical stability window of a polymer electrolyte film is important, since it must work at a wide range of voltage for its practical applications. The cyclic voltammogram curves for 60PVA-40LiTFSI + *x* EMITFSI (*x* = 0, 5, 10, and 15 wt.%) films, recorded over the voltage range of −3.0 to 3.0 V at room temperature, are shown in Figure 6c (SS/P-40-*x*/SS cells). With the addition of ionic liquid EMITFSI, the electrochemical stability window of the film gradually widens, and the current fluctuation gradually becomes small. However, for the P-40-15 system, the current fluctuation becomes apparent again, probably due to the superfluous ionic liquid. The electrochemical stability window of the P-40-10 film is as wide as ~5 V, which is good enough for electrochemical device applications.

Based on the above results, the 60PVA-40LiTFSI + 10EMITFSI (P-40-10) system was chosen as both the separator and electrolyte to fabricate the EDLCs so as to demonstrate the validity of the P-40-10 electrolyte system. To investigate the performance of EDLCs, cyclic voltammetry (CV) and galvanostatic charge-discharge (GCD) cycling were performed in the potential ranges of 0–1.6 V, 0–2 V, 0–2.4 V, and 0–3 V at room temperature (~25 °C). Finally, the assembled EDLC was used to power a light-emitting diode (LED).

**Figure 7:**
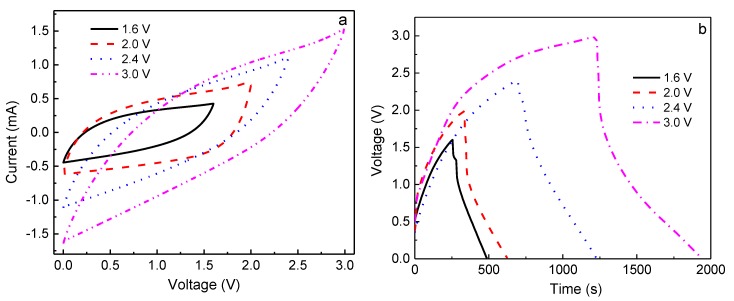

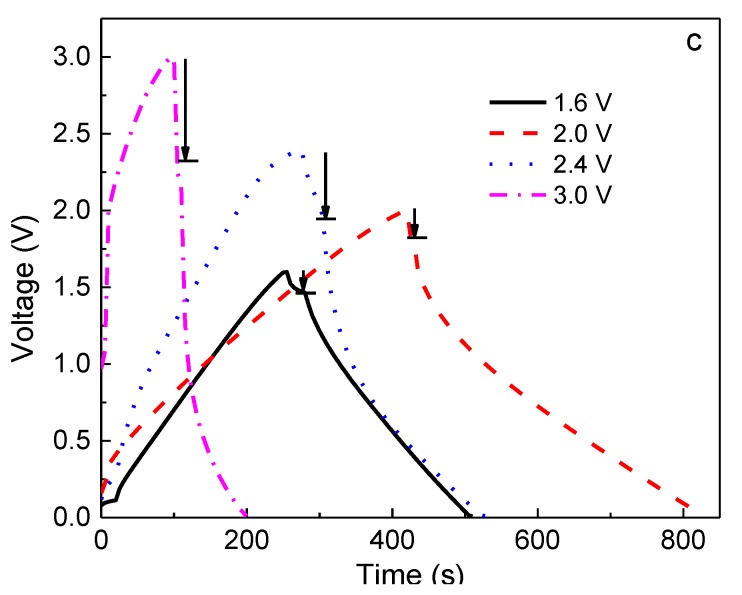
(**a**) Cyclic voltammetry curves at a scan rate of 5 mV s^−1^, (**b**) charge-discharge curves of 1st cycle, and (**c**) charge-discharge curves of 100th cycle, for the electric double layer capacitor (EDLC) fabricated with the P-40-10 electrolyte system, performed at a current density of 0.4 A g^−1^ under different working voltages.

Figure 7a depicts the CV plots of the EDLC at different working potentials, and Figure 7b,c shows the constant current density (0.4 A g^−1^) charge-discharge curves at different working potentials for the 1st and 100th cycles. It is clearly seen from Figure 7a that the CV plots show relatively perfect rectangular shapes at the potential ranges of 0–1.6 and 0–2 V, indicating the rapid current response to the voltage changed at the two ends of the potential range, and the relatively ideal capacitive behavior. Nevertheless, the curve shape becomes slightly leaf-like, but without a visible redox peak, as the charging potential increases to 2.4 and 3.0 V, implying that the characteristic of a capacitor still exists at these high voltages; that is, the charge and discharge could reversibly take place at the electrolyte-electrode interface [35]. Furthermore, the enclosed area by the CV curve becomes bigger with increasing working voltage, indicating that the larger specific capacitance of EDLC is expected at a higher charging voltage [36]. In order to confirm this, the galvanostatic charge-discharge cycling of the EDLC at various working voltages is also investigated, as shown in Figure 7b,c. Obviously, with increasing working voltage, the symmetric characteristic of charge-discharge plots gradually deviates, meaning that the performance of the EDLC at a higher voltage becomes worse, which is in accordance with the results of CV. Moreover, the discharge time for the 1st cycle becomes longer with increasing working voltage (Figure 7b), indicating that a larger specific capacitance (*C*_s_) can be obtained at a higher charging voltage, which is given by [18]

(5) $$C_{s}=4I/(m\frac{dV}{dt})\;$$

where *I* is the current, *m* is the total mass of electrodes, and d*V/*d*t* is the slope of the fitting straight line to the discharge curve. The *C*_s_ of the EDLC obtained for the 1st cycle was 103, 107, 143, and 164 F g^−1^ at 1.6, 2.0, 2.4, and 3.0 V, respectively. However, it is observed that the discharge time shortens when the charging voltage exceeds 2 V after 100 cycles (Figure 7c), demonstrating that the specific capacitance becomes lower, being *C*_s1.6V_ = 103 F g^−1^, *C*_s2.0V_ = 138 F g^−1^, *C*_s2.4V_ = 69 F g^−1^, and *C*_s3.0V_ = 32 F g^−1^. This phenomenon can be explained by the ion blocking effect in the electrolyte and the dead volume effect in electrodes. In general, more charge carriers in electrolyte can be created when a higher charging voltage is applied to an EDLC, leading to more stored charges in electrodes, thereby leading to a higher initial specific capacitance. However, the ion blocking effect will appear after several cycles, enhancing internal resistance (*IR*, *IR*_1.6_ < *IR*_2.0_ < *IR*_2.4_ < *IR*_3.0_), especially in terms of the large size of TFSI^−1^ (0.8 nm), which is close to the pore size of activated carbon material (~1 nm) [37,38], resulting in a larger dead volume in electrodes and thus a lower specific capacitance.

**Figure 8:**
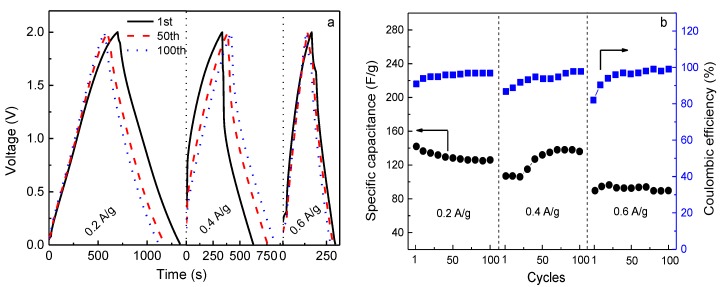
(**a**) Charge-discharge curves of 1st, 50th, and 100th cycles at different current densities under a charging voltage of 2 V for the EDLC fabricated with the P-40-10 electrolyte system, and (**b**) the corresponding cycling durability over 100 cycles for each current density.

In order to fulfill the requirements of practical applications, the rate capability of the EDLC was examined by charge-discharge cycling at different current densities, as shown in Figure 8a. The corresponding values of specific capacitance and coulombic efficiency are represented in Figure 8b. The EDLC is charged from 0 to 2.0 V and discharged from 2.0 to 0 V at ambient temperature. It is seen that the discharge behavior for each current density is almost linear, implying the capacitive characteristic of the capacitor. Additionally, the charge-discharge plots are symmetric after some cycles. This means that there should be a high coulombic efficiency (*η*). *η* is an important parameter as it is correlated to the cycling stability of the capacitor, which is given by [18]

(6) $$\eta =\frac{t_{D}}{t_{C}}\times 100\%\;$$

where *t*_D_ is the discharge time and *t*_C_ is the charge time. As shown in Figure 8b, the values of *η* for the EDLC are 91%, 87%, and 82% in the first charge-discharge cycle at the current densities of 0.2, 0.4, and 0.6 A g^−1^, respectively. *η* decreases with increasing current density. This is because at a low charging rate the ions have enough time to adsorb to, or desorb from, the vacant sites in electrode material, resulting in a high initial specific capacitance [39] (*C*_s0.2A/g_ = 142 F g^−1^, *C*_s0.4A/g_ = 107 F g^−1^, and *C*_s0.6A/g_ = 90 F g^−1^). After some cycles, the coulombic efficiency at all the charging rates is almost 100%, as all ions adsorbed to vacant sites can completely desorb. Meanwhile, the specific capacitance of the EDLC charged at 0.4 A g^−1^ increases and approaches the initial *C*_s_ obtained at 0.2 A g^−1^, possibly because the ions can accommodate the electric field density and sufficiently utilize the vacant sites in electrodes. However, it decreases with further increasing current density, which is a common phenomenon for EDLCs [40]. Obviously, the value of *C*_s_ at 0.6 A g^−1^ is lower, but the capacitance retention is still ~100%.

**Figure 9:**
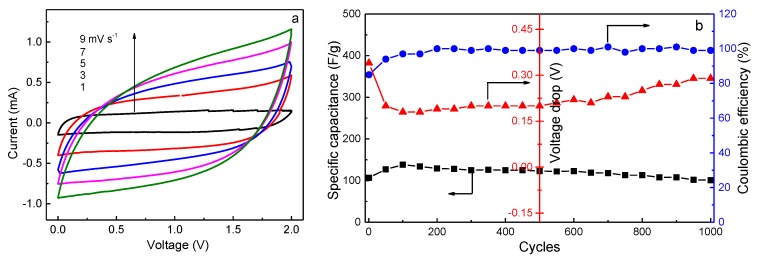
(**a**) Cyclic voltammetry (CV) curves at different scan rates in the potential range of 0–2 V, (**b**) cycling durability at 0.4 A g^−1^ over 1000 cycles for the EDLC fabricated with the P-40-10 electrolyte system.

Cyclic voltammetry characteristics of an EDLC provide information about the nature of charge storage at the interfaces in the cathodic and anodic regions [39,41]. The CV plots of the present EDLC, recorded in the potential range of 0–2 V at different scan rates, are shown in Figure 9a. An ideal square shape of the CV curve without any visible redox peaks persists until the scan rate rises to 7 mV s^−1^, implying the free diffusion of ions at a constant rate, and the formation of double-layers at the interfaces. Nevertheless, it is seen that the rectangle has a slight deviation as the scan rate further increases. This phenomenon is due to the equivalent series resistance (*ESR*) of the EDLC, which includes the resistance between the current collector and the electrode, the intrinsic resistance of the electrode, the resistance at the electrode-electrolyte interface, and the bulk resistance of the electrolyte [38]. *ESR* can be calculated by [42]

(7) $$\;ESR\;=\;\frac{V_{\mathrm{drop}}}{2i}\;$$

where *i* is the constant current and *V*_drop_ is the voltage drop on discharging. The effect of *ESR* on EDLC can be explained on the basis of Figure 9b. When the EDLC is charged from 0 to 2.0 V at a current density of 0.4 A g^−1^, *ESR* has the highest value in the first cycle because the voltage drop is the largest, resulting in a lower initial coulombic efficiency and specific capacitance. *V*_drop_ initially decreases and then increases with increasing charge-discharge cycles, leading to the opposite variation of specific capacitance. However, it is noted that *C*_s_ is nearly constant over 1000 cycles, implying its perfect cycling stability. The long cycle lifetime of EDLC is one of the important criteria for its good performance. In addition, both the energy density (*E*_cell_, W h kg^−1^) and the power density (*P*_cell_, W kg^−1^) are crucial parameters because they ensure the practical application of an EDLC device. *E*_cell_ and *P*_cell_ can be calculated by [43]

(8) $$E_{\mathrm{cell}}=\frac{C_{s}{\left(\Delta V\right)}^{2}}{8}\times \frac{1000}{3600}\;$$

(9) $$P_{\mathrm{cell}}=E_{cell}/\left(\frac{t_{D}}{3600}\right)\;$$

where Δ*V* is the charging voltage minus the voltage drop (*V*_drop_). The values of *C*_s_, *E*_cell_, and *P*_cell_ in the 1st and 1000th cycles are listed in Table 3. It is seen that high capacity retention is obtained over 1000 cycles, and the energy and power densities remain almost unchanged after cycling, which is beneficial to the practical application of EDLC. Lim et al. [35] reported that the *C*_s_ value of EDLC fabricated with PVA-LiClO_4_-TiO_2_ electrolyte (*σ* = 1.3 × 10^−4^ S cm^−1^) and activated carbon electrodes is 12.5 F g^−1^ after 1000 cycles when charging at a constant current of 1 mA under 1.0 V. Obviously, the present value is much higher than this one.

**Figure 10:**
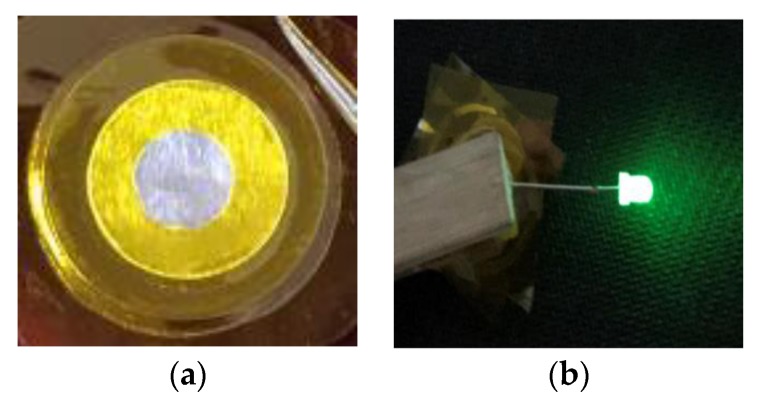
(**a**) Digital photo of EDLC fabricated with the P-40-10 electrolyte system, (**b**) powering a light-emitting diode (LED) with EDLCs connected in series using conductive silver paste.

To demonstrate the applicability of the P-40-10 electrolyte system, the EDLC fabricated with this electrolyte (as shown in Figure 10a) was used to power a light-emitting diode (LED), as shown in Figure 9b. It is worth mentioning that since the voltage rating of the LED is ~3.4 V, the device is composed of two EDLCs charged to 2.0 V and connected in series using conductive silver paste, which is glued to the uncovering aluminum foil. Obviously, the LED emits extremely dazzling green light. This further confirms the excellent electrochemical performance and practical utility of PVA-LiTFSI-EMITFSI electrolytes.

## 4. Conclusions

High ionic-conductivity gel polymer electrolyte films based on biodegradable PVA-LiTFSI-EMITFSI along with 1-methyl-2-pyrrolidinone as the solvent were successfully prepared. The gel polymer electrolyte films are capable of coordinating and transporting Li^+^ ions, and have a relatively wide electrochemical stability window (~5 V), which is good enough for Li-ion device applications. Moreover, the 60PVA-40LiTFSI + 10 wt.% EMITFSI film exhibits excellent mechanical properties. The relaxation time of the film is as short as 5.30 × 10^−7^ s, indicating a large quantity of amorphous domains in the film, and a high ionic mobility. Hence, the room-temperature ionic conductivity of the film reaches a high value of ~3.6 × 10^−3^ S cm^−1^. This film was applied in electric double-layer capacitors (EDLCs). The EDLC has a specific capacitance of 101 F g^−1^ and an energy density of 10.3 W h kg^−1^, even after 1000 charge-discharge cycles at a current density of 0.4 A g^−1^ under a charging voltage of 2 V, implying that the specific capacitance and energy density retentions are as high as 94.4% and 98.1%, respectively. All these values demonstrate that the 60PVA-40LiTFSI + 10 wt.% EMITFSI film is a promising electrolyte candidate for electronic device applications.

## Figures and Tables

**Table 1:** Values of relaxation time for the films.

Samples	Relaxation Time (s)
P-40	6.38 × 10^−6^
P-40-5	6.50 × 10^−6^
P-40-10	5.30 × 10^−7^
P-40-15	7.90 × 10^−6^

**Table 2:** Mechanical properties of the films.

Samples	Young’s Modulus (MPa)	Yield Strength (MPa)	Breaking Strain (%)
Pure PVA	178.0	17.5	500
P-40	42.6	9.0	710
P-40-10	26.6	6.2	1130

**Table 3:** Specific capacitance, energy density, and power density of the EDLC at a current density of 0.4 A g^–1^ under a working voltage of 2 V.

Performance	1st Cycle	1000th Cycle
*C*_s_ (F g^−1^)	107	101
*E*_cell_ (Wh kg^−1^)	10.5	10.3
*P*_cell_ (W kg^−1^)	132.6	132.9
